# Why Defective Nasal Respiration Impedes Growth and Development

**Published:** 1906-03

**Authors:** P. Watson Williams

**Affiliations:** Lecturer on Diseases of the Nose and Throat, University College, Bristol; Physician in charge of the Throat Department, Bristol Royal Infirmary


### WHY DEFECTIVE NASAL RESPIRATION IMPEDES GROWTH AND DEVELOPMENT.

BY

P. Watson Williams, M.D. Lond.,

*Lecturer on Diseases of the Nose and Throat, University College, Bristol; Physician in charge of the Throat Department, Bristol Royal Infirmary.*

Defective nasal respiration is not synonymous with nasal stenosis, and many mouth-breathers have no obstruction in the nasal passages sufficient to prevent them breathing through the nose. Why and how imperfect nasal respiration becomes a source of ill-health and of defective development is worthy of consideration, if only as a guide to treatment for remedying the habit, which often persists after actual obstruction has been removed, and may exist without any obstruction sufficient to necessitate operative measures.

The evil effects of mouth-breathing are due (1) to the inspired air not being filtered, warmed and saturated with moisture before reaching the lower air passages; (2) the constant effort to inspire through a partly blocked nose; (3) the absence of the stimulation to respiration that normal nasal respiration affords.

It has been calculated that at least 1,500 organisms are inhaled into the nose every hour, while it must be a common event, according to St. Clair Thomson and Hewlett, for 14,000 to pass into the nose in a single hour in an average London atmosphere. Nevertheless, the expired air is practically germ free, nearly all the organisms in the inspired air being arrested in the nasal passages. There must be a considerable number of pathogenic organisms amongst those inhaled in any city atmosphere, hence we find that in any diphtheritic epidemic a large number of healthy children may yield diphtheria organisms on cultures being taken from the nose; and if, as has sometimes been done, these are considered to be infected with diphtheria on cultural evidence alone, quite uncalled-for alarm may arise and a number of healthy individuals be unnecessarily certified as suffering from diphtheria. So too with other organisms, such as the meningococcus, and could we as readily identify the organisms of scarlatina, and other common infectious diseases, we should doubtless find them in the noses of healthy individuals.

Then, again, it has been repeatedly demonstrated that the secretory functions of the nose vary in activity with the needs of the moment, and no matter how dry the atmosphere may be, the inspired air becomes completely or almost completely saturated with moisture as it passes through the nasal passages, and is at the same time raised in temperature to normal blood heat. It has been estimated that about half a pint of water is taken up by the inspired air in the course of twenty-four hours, and, if the air is to reach the lungs in a normal condition of saturation, it is impossible for that amount of moisture to be taken up from the mucous membrane of the throat without serious risk to its physiological integrity. Incidentally we may refer to the still too prevalent idea that dry air, such as that of the higher Alpine health resorts, exercises a beneficial effect on pulmonary diseases because it is dry. An obvious fallacy, inasmuch as even this dry air becomes saturated with moisture before it reaches the lung, and if from any defect in the nose this does not occur, the very dryness becomes a fruitful source of tracheal and bronchial trouble.

The pernicious effects of mouth-breathing in children are seen in the constant tendency to infective catarrhs, bronchial colds and pulmonary complaints from which children with adenoids and some other causes of nasal obstruction are so prone to suffer.

The mechanical or physical effects due to the effort of a child to breathe through partially stenosed nasal passages without doubt accounts for much of the secondary pathological consequences of nasal obstruction, especially the defective expansion of the lungs and deformity of the chest walls. Personally, I am convinced that the mechanical factor, though undoubtedly potent, has been exaggerated, and that other important physiological factors have been overlooked.

While not by any means the only source of defective nasal respiration in childhood, it may be conceded that by far the commonest cause is the presence of "adenoid growths"; yet in a large proportion of these patients the adenoid growths do not cause any actual mechanical obstruction in the nasal passages, and in all cases I am convinced that it is rather the indirect results of adenoids which are mainly responsible for the abnormal respiration. Hence we find that the pernicious effect of relatively small amounts of adenoid growth may be as marked or even more pronounced than in some patients with large adenoid growth. It was formerly believed that enlarged tonsils caused pigeon-breast by mechanical impediment to free inspiration, but when we remember how much smaller is the glottic chink in a child than is the available air space between almost any case of chronically enlarged tonsils, it is obvious that the narrower airway is within the normal larynx. Now, however, it is held that the co-existing adenoid growths are responsible for the chest defcrmities, and though in cases where these growths are large they offer mechanical obstruction to inspiration, in a large number of children, who suffer equally, there is no mechanical obstruction comparable to the narrow normal glottic chink. These minor degrees of adenoids, as do all adenoid growths, maintain a more or less persistent or frequently recurring nasal catarrh, which is really the most potent cause of defective respiration, just as catarrh of the Eustachian tube, and not mechanical occlusion of the Eustachian orifices by the growths themselves, causes the deafness which is so constantly associated with adenoid growths. Thus we may find an explanation of those cases of partial but purely mechanical obstruction to nasal respiration, *e.g.* by anterior deflections of the septum, or the rarer partial occlusion of the nasal passages at the inner extremity of the vestibule, in which, unless adenoids co-exist, the chest deformities may be absent, despite the equally marked defective patency of the nasal passages. In a sense, every child has some adenoid growth in the naso-pharynx, for a considerable aggregation of healthy lymphoid tissue is the birthright of every child, as is a pair of healthy faucial tonsils; the removal of this healthy naso-pharyngeal tonsil is not a proof that the child had adenoids.

Thus it is the secondary or indirect results of unhealthy lymphoid tissue in the naso-pharynx, the consequent persistent nasal catarrh which the adenoids keep up, which is very largely responsible for the defective nasal respiration, and it is the absence of normal nasal respiration which causes the pulmonary troubles and defective development of the chest wall so generally present. Other conditions such as marked septal deflection, hypertrophy of the turbinated bodies, accessory sinus disease, polypi and disease of the ethmoidal labyrinth, congenital occlusion of the nasal passages anteriorly or at the posterior choanæ, if sufficient to markedly impede nasal respiration, are effective, though less potent factors impeding growth and development in children. Why?

Under normal conditions breathing is an involuntary act, for though it may be temporarily arrested or accelerated by an effort of the will, it is habitually maintained and regulated according to the degree of venosity of the blood by the respiratory centre in the medulla, and the activity of external respiration—*breathing*—depends on and corresponds with the activity of internal respiration—*tissue metabolism*—thus if a child runs he breathes more rapidly.

But although the respiratory centre is largely automatic, its activity is influenced by afferent impulses, and therefore is to a certain extent a reflex centre. Thus there is ample evidence that the normal activities of the respiratory centre depend on impulses reaching it from the lung through the vagi, from the surface of the skin, and lastly, but not least, from the nasal and naso-pharyngeal passages through the fifth and glosso-pharyngeal nerves.

That stimulation of the upper part of the nasal passages influences respiration has been demonstrated experimentally by Spencer, who likewise obtained slowing and acceleration of the respiration rate from stimulation of the olfactory limb of the anterior commissure and of the area around the upper end of the infra-orbital sulcus respectively, while common experience tells us that irritation of the nasal passages by smelling salts influences respiration, and intense subjective sense of dyspnœa following the insertion of the finger in the naso-pharynx, a method still used for the diagnosis of adenoids, is due to the same respiratory inhibitory impulse that sometimes causes a dangerous arrest of respiration during the operation for the removal of adenoids under anæsthesia. Thus we find in the absence of normal nasal respiratory stimuli, with the existence of adenoids in the naso-pharynx, a cause for the deficient activity of the respiratory centres in young growing children, which results in infra-clavicular flattening of the chest wall.

I should be sorry to under-estimate other factors which must be held accountable for the defective physiological activity of the respiratory centres in children suffering from enlarged tonsils and adenoids, chief among which is a chronic sapræmia, causing tissue inactivity and hence absence of the normal need for more rapid respiratory exchanges, but I am anxious to emphasise the physiological import of the nose in influencing respiratory activity, and the pernicious effect of the absence of nasal respiration, not alone from the commonly recognised results of unwarmed and unmoistened air reaching the lungs, but from the persistent abrogation of nasal respiratory stimulation. The benefit which is anticipated from the removal of adenoids may fail to be realised either because the real cause of nasal obstruction has been overlooked or only partly removed, or because the habit of breathing through the nose has persisted so long that, without suitable respiratory exercises and general hygienic treatment, the normal factors are not brought into play. It is scarcely an exaggeration to say that children who persistently fail to duly expand their lungs, in other words, whose respiratory exchanges are subnormal, are underfed, for a due supply of inspired oxygen is essential for the utilisation of food in the processes of "internal respiration."

Disappointing results may follow operative treatment in nasal obstruction, firstly, because the real cause of the obstruction has been overlooked, (generally because intra-nasal obstruction has been wrongly diagnosed as adenoids) ; or, in the second place, because the original cause of defective nasal respiration having been removed, the necessary measures for re-establishing long-lost normal respiration are not applied.

Physiological conditions do not always persist in their entirety despite abnormal states, and thus it is that pathological conditions in the nose, apart altogether from the mechanical obstruction which may or may not exist in any material degree, become associated with diminished demand for, and hence diminished respiratory exchange. "Forced" feeding will increase nutrition, provided, of course, assimilation can be assured, and so, too, respiratory exercises properly applied improve deficient respiration and increase the physiological demand for a fuller respiratory exchange; and if anyone doubts the possibility of increasing a healthy "air-hunger" as well as inducing a normal gastric appetite, let him meet some healthy patients fresh from a sanatorium for consumption.

After the removal of any abnormal conditions in the nose and throat, one should advocate open-air methods, cold bathing, and particularly physiological drill comprising appropriate respiratory exercises, in order that these listless, partially asphyxiated children should grow up in the fulness of life.

---

### SOME EFFECTS OF FRIGHT IN MEDICINE.[1]

